# Correction: Water resource management: IWRM strategies for improved water management. A systematic review of case studies of East, West and Southern Africa

**DOI:** 10.1371/journal.pone.0304228

**Published:** 2024-05-17

**Authors:** Tinashe Lindel Dirwai, Edwin Kimutai Kanda, Aidan Senzanje, Toyin Isiaka Busari

The affiliations for the first author is incorrect. Tinashe Lindel Dirwai is not affiliated with #2.

The affiliation for the third author is incorrect. Aidan Senzanje is not affiliated with #1 but with #2: School of Engineering, University of KwaZulu-Natal, P.Bag X01, Pietermaritzburg, South Africa.
