# Supplementary material for: Water resource management: IWRM strategies for improved water management. A systematic review of case studies of East, West and Southern Africa
Source: PLoS One. 2021 May 25;16(5):e0236903. doi: 10.1371/journal.pone.0236903 (PMC8148310; doi:10.1371/journal.pone.0236903)
Supplement: S1 Table — (DOCX) [file pone.0236903.s002.docx]

**S1 Table. Data extracts with the applied codes.**

| Region | Driver | Data Extract | Coded for |
| --- | --- | --- | --- |
| East Africa | Water scarcity | Kenya is a water-scarce country with per capita water availability of 586 m^3^ in 2010 and projected to 393 m^3^ in 2030 (Kibiiy and Kosgei, 2018). | Population growth |
|  |  | Uganda is endowed with water resources; however, it is projected that the country will be water-stressed by 2020 which could be compounded by climate variability and change, rapid urbanization, economic and population growth (Kilimani et al., 2015). | Population growth and industrial expansion |
|  | Trans-boundary Water Resources | The Nile River system is the single largest factor driving the IWRM in the region. Lake Victoria, the source of the Nile River is shared by the three East African states of Kenya, Uganda and Tanzania. | Shared water sources |
|  |  | The Mara River is another trans-boundary river which is shared between Tanzania and Kenya and the basin forms the habitat for the Maasai Mara National Reserve and Serengeti National Park in Kenya and Tanzania | Ecological |
|  | Donor Influence | The World Bank has been pushing for IWRM principles through the Nile Basin Initiative (NBI) | World Bank aid throughtransboundary initiatives |
|  |  | Structural Adjustment Programmes (SAPs) introduced by the World Bank and International Monetary Fund (IMF) | Structural Adjustment Programmes |
|  |  | Allouche [5] pointed that Uganda had become a ‘darling’ of the donor countries in the early 1990s and that DANIDA helped to develop the Master Water Plan and the country was keen to show a willingness to develop policy instruments favourable to the donor. | Latent donor influence |
| West Africa | Water scarcity | Countries in the Sahelian regions are characterised by semi-arid climatic conditions. | Arid climate |
|  | Government Intervention and Regional Bloc Pro-Activity | The Burkinabe government exhibited political goodwill such that in 1995 the government brought together two separate ministries into one ministry of Environment and Water thus enabling coherent policy formulation and giving the ministry one voice to speak with on-water matters. | Policy coherence |
|  | Trans-boundary Water Resources | West Africa has 25 transboundary watercourses and only 6 are under agreed management and regulation | Shared water sources |
|  |  | Unregistered rules and the asymmetrical variations associated with watercourses warrant the introduction of the IWRM principle to set equitable water sharing protocols and promote environmental flows (e-flows). The various acts signed represent an evolutionary treaty development that combines the efforts of riparian states to better manage the shared water resources. | Ecological and equitable water sharing |
|  | Donor Influence | Donor aid cannot be downplayed in pushing for policy diffusion in low-income aid-dependent countries. GoBF [47] cites that from the period 1996 – 2001, more than 80% of water-related projects were donor funder. | Donor effect |
|  |  | Research by Cherlet and Venot [48] also cites that almost 90% of the water investments in Mali were funded outside the government apparatus. | Donor effect |
|  | Pro-active Citizenry | Burkina Faso and Mali’s adoption story is accentuated by heightened agency, the individual enthusiasm on having an influence on the outcome facilitated policy diffusion and can be argued to be a potential innovation diffusion driver for the IWRM policy approach in the region. | Pro-active citizenry |
| Southern Africa | Government policy | In 1998 a National Water Policy (NWP) which was in alignment with the Dublin principles was enacted, thus giving Zimbabwe an IWRM policy footing. | Policy formulation and adoption |
|  |  | The Fast Track Land Reform (FTLR) programme disaggregated the large-scale commercial farms and created smallholder farming [51], consequently influencing and dictating IWRM policy path. | Radical innovation |
|  |  | Continued clashes of water and land reforms have created laggards in the IWRM adoption. | Silo effect |
|  |  | IWRM suffered another shock caused by the governing party when they introduced radical innovations that sought to shift from the socialist to neoliberal water resource use approach. The radical innovation through the government benefited the large-scale commercial farmers at the expense of the black smallholder farming community. | Radical innovation |
|  | Donor Influence | A lack of access to international funding and fleeting donor aid exacerbated the policy uptake as such the anticipated implementation, operationalisation and continuous feedback mechanism for policy revision and administering process was never realised. | Donor unavailability |
|  | Policy misinterpretation | The shift from Integrated Catchment Management (ICM) to IWRM hindered the operationalisation and diffusion of the IWRM practice.  IWRM diffusion encountered obstacles partly because the government lacked capacity to ensure policy cascading. | Policy misinterpretation |
|  | Wet Water and Paper Water | The shift from local water rights system to state-based water system have created bottlenecks making it hard for smallholder farmers to obtain “paper water” and subsequently “wet water” | Inflexible policy |
